# An education program for infection link staff in three Danish hospitals

**DOI:** 10.1186/1753-6561-5-S6-P282

**Published:** 2011-06-29

**Authors:** A Juhl-Jorgensen, A-M Thye

**Affiliations:** 1 Clinical Microbiologi, Hillerod Hospital, Hillerod, Denmark

## Introduction / objectives

Prevention and control of Healthcare Associated Infections is a priority in the ”Danish Quality Model for Public Health”, and is part of the accreditation requirements from the Joint Commission International.In order to reduce the number of hospital acquired infections we trained 100 persons from all departments of the three local hospitals as Infection Link Staff.

## Methods

- Use of an education program that is elementary and targeted to the every day running of each department

- Training of at least one person from all the clinical departments

- Holding a training seminar in infection prevention for all Infection Link Staff on three non-consecutive days with an observation study phase between day 2 and 3

## Results

Infection prevention has been brought emphatically into focus. Department leadership and staff are satisfied and hygienic behavior is improving. It still is too early to observe the hoped for reduction of hospital acquired infections.

## Conclusion

The education program needs to be basic and target the every day running of each department and the training of at least one person in all the clinical departments is important so that infection prevention measures can be assured on a daily basis. It is imperative that Infection Link Staff have the full support of Departmental leadership and it requires a sustained effort from the Hygiene Unit to maintain the standard of the Infection Link Staff, as this can easily become victim to sick leaves, overwork, etc.

Continuous education of the Infection Link Staff in the form of networking, receipt of information, participation in seminars, etc. is a must and spread of the concept of Infection Link Staff education to other hospitals is desirable as it appears to be a good way of implementing infection control.

## Disclosure of interest

None declared.

